# Breast Cancer Molecular Subtype Prediction: A Mammography-Based AI Approach

**DOI:** 10.3390/biomedicines12061371

**Published:** 2024-06-20

**Authors:** Ana M. Mota, João Mendes, Nuno Matela

**Affiliations:** 1 Instituto de Biofísica e Engenharia Biomédica, Faculdade de Ciências, Universidade de Lisboa, 1749-016 Lisbon, Portugal; jpmendes@fc.ul.pt (J.M.); nmatela@ciencias.ulisboa.pt (N.M.); 2 LASIGE, Faculdade de Ciências, Universidade de Lisboa, 1749-016 Lisbon, Portugal

**Keywords:** breast cancer, molecular subtypes, mammography, artificial intelligence, deep learning, personalized medicine

## Abstract

Breast cancer remains a leading cause of mortality among women, with molecular subtypes significantly influencing prognosis and treatment strategies. Currently, identifying the molecular subtype of cancer requires a biopsy—a specialized, expensive, and time-consuming procedure, often yielding to results that must be supported with additional biopsies due to technique errors or tumor heterogeneity. This study introduces a novel approach for predicting breast cancer molecular subtypes using mammography images and advanced artificial intelligence (AI) methodologies. Using the OPTIMAM imaging database, 1397 images from 660 patients were selected. The pretrained deep learning model ResNet-101 was employed to classify tumors into five subtypes: Luminal A, Luminal B1, Luminal B2, HER2, and Triple Negative. Various classification strategies were studied: binary classifications (one vs. all others, specific combinations) and multi-class classification (evaluating all subtypes simultaneously). To address imbalanced data, strategies like oversampling, undersampling, and data augmentation were explored. Performance was evaluated using accuracy and area under the receiver operating characteristic curve (AUC). Binary classification results showed a maximum average accuracy and AUC of 79.02% and 64.69%, respectively, while multi-class classification achieved an average AUC of 60.62% with oversampling and data augmentation. The most notable binary classification was HER2 vs. non-HER2, with an accuracy of 89.79% and an AUC of 73.31%. Binary classification for specific combinations of subtypes revealed an accuracy of 76.42% for HER2 vs. Luminal A and an AUC of 73.04% for HER2 vs. Luminal B1. These findings highlight the potential of mammography-based AI for non-invasive breast cancer subtype prediction, offering a promising alternative to biopsies and paving the way for personalized treatment plans.

## 1. Introduction

Cancer is among the four top causes of premature death under 70, with breast cancer being the most diagnosed and one of the deadliest among women, with an estimated 2.3 million new cancer cases (one in four new cancer cases) and 685,000 cancer deaths (1 in 6 deaths) in 2020 [1]. Approximately half of all breast cancers occur in women with no specific risk factors other than gender and age.

Early diagnosis, typically achieved through generalized screening programs, is crucial for improving prognostic outcomes [2]. However, despite the benefits of early diagnosis, the clinical presentation of the breast cancer, its response to therapy, and its prognosis are highly dependent on its molecular characteristics [3].

Most breast cancers are carcinomas and can be subdivided into in situ carcinoma or invasive carcinoma. While the first is a non-invasive or pre-invasive type of breast cancer, in the second, the cancer cells have grown into the surrounding breast tissue. Invasive breast carcinoma is the classic and most common type of breast cancer and poses a higher risk of metastasis, requiring more aggressive treatment compared to in situ carcinoma, which typically necessitates surgical excision and close monitoring to prevent progression to invasive disease.

The molecular classification of invasive breast carcinoma according to St. Gallen International Consensus Guidelines from 2013 [4] comprises four main intrinsic molecular subtypes based on the expression of immunohistochemical markers, including estrogen (ER) and progesterone (PgR) receptors, the detection of the overexpression and/or amplification of the human epidermal growth factor receptor 2 (HER2) oncogene and the protein marker Ki-67, which is an indicator of cell proliferation. When any of the hormone receptors are positive, the breast cancer is classified as Luminal. The Luminal classification is further divided into Luminal A and Luminal B. Luminal A is characterized by being HER2 negative with a Ki-67 level below 14%. Luminal B can be divided into two categories: either having the PgR receptor negative or Ki-67 level above 14% (Luminal B1) or being positive for HER2 (Luminal B2). If the tumor is negative for both hormone receptors but there is an overexpression of HER2, then it is classified as HER2-enriched (“HER2”) and if it is negative for ER, PgR and HER2, it is classified as basal-like, also known as “Triple Negative” (“TN”). This classification is summarized in the Table 1.

Luminal A cases represent 70% of all diagnoses and tend to be less aggressive than Luminal B (about 10%), which is usually associated with a worse prognosis. HER2-enriched cancers are the least common subtype, accounting for nearly 5% of diagnoses, though outcomes for this type have been improving over the years due to innovative targeted therapy. Finally, TN breast cancers represent 11% of diagnosed cases and are very aggressive, with a high risk of metastasis and recurrence [5,6].

The identification of the molecular subtype of the breast cancer is of extreme importance to define the line of treatment to pursue. Currently, in order to obtain histological and molecular characterization of the tumor, a biopsy is always performed, and the extracted biological material is analyzed using pathological histology techniques. This is a very specialized procedure, quite expensive and time-consuming, often yielding results that have to be supported by additional biopsies due to error on the technique procedure and/or the heterogeneity of the tumor. In addition, performing a biopsy presents several possible complications, such as bleeding or infections [7]. Molecular profiling, genomic Tests (e.g., Oncotype DX and MammaPrint), proteomics and metabolomics further refine tumor characterization but also rely on biopsy-derived samples for detailed analysis of genetic, protein, and metabolic profiles [8,9,10].

Breast imaging with mammography is non-invasive and provides information about the entire tumor and its microenvironment, information that is not provided by a traditional biopsy. With this data, some characteristics of the tumor, such as its overall shape, heterogeneity, or growth/regression over time, can be assessed. Furthermore, it is known that the imaging characteristics of each tumor are strongly related to the different molecular subtype [11]. This means that some histological information about the tumor can be accessed with a non-invasive procedure directly from the image.

Recently, the increase in computational power and the availability of bigger imaging datasets have enabled the development of successful deep learning artificial intelligence (AI) algorithms in medical imaging. In fact, there are some recently published works addressing the use of AI algorithms to predict the breast cancer molecular subtype in mammography images [12,13,14]. Wang et al. [12] aimed to differentiate TN from non-TN tumors using radiomics. Using a dataset of 51 cases (23 TN, 31 non-TN), the authors extracted 396 features of the segmented tumors and employed the maximum relevance minimum redundancy (mRMR) algorithm and the least absolute shrinkage and selection operator (LASSO) method to eliminate redundant and irrelevant features. Ultimately, three features were used to differentiate TN from non-TN cases. The authors report an AUC of 0.84; however, the number of features used is very sparse as the number of cases being analyzed is substantially low. Any conclusions that could be drawn from these results are very restricted due to the presented limitations.

The same goal was addressed by Ge et al. [13]. In this work, the “verification” set used by the authors has a larger number of subjects, with 108 cases. Even though there is a substantial increase in the number of instances being classified, the obtained results (AUC = 0.809) need to be looked upon carefully. When exploring this verification set, it is possible to verify that from the 108 cases, only 25 (<25%) are TN cases. This discrepancy is also observed in the training set, where less than 20% of the cases are TN, potentially impairing the capability of the model to identify the positive class. In fact, that impairment can be seen when analyzing the positive predictive value metric, which is near 55%. 

Deng et al. [14], on the other hand, aimed to classify HER2-positive cases using radiomics. While there was substantial data imbalance in all image sets used, the authors countered this limitation by using an oversampling approach. The most relevant radiomic features were used to develop a Gradient Boosting Machine model, which achieved an AUC of 0.776 on the test set.

There are two main limitations regarding the mentioned studies: either the authors use a standard radiomic approach, which limits tumor analysis to handcrafted features and/or the training/test set has a very small sample size (<100 images), limiting the conclusions that can be drawn from the study.

Considering the presented limitations of the radiomics approaches, two very recent studies suggested the use of a larger sample size and the consideration of deep features for classification purposes instead of restricting the analysis to handcrafted features. Nissar et al. [15] proposed an attention-based deep learning model to make several predictions about breast cancer in mammograms, including the molecular classification of lesions into four classes: TN, HER2-positive, Luminal A, and Luminal B. While the proposed methodology achieves impressive results, with an accuracy of 86%, critical information about the test set used is missing. The full sample size comprises 2358 images before augmentation and 4987 after augmentation. However, details about class distribution and the division of data into training, validation and test sets are not provided. Additionally, it is unclear whether augmented images were used in both the training and testing sets. These limitations inhibit the drawing of definitive conclusions about the model’s effectiveness. Qian et al. [16] also used lesion attention-based to do biomarker status prediction on contrast-enhanced mammography. Specifically, regarding the identification of HER2 status (HER2 vs. non-HER2), the authors used a test set of 152 images, with approximately 28% of them belonging to the positive class and the remaining to the negative class. This approach achieved an AUC of 0.67, with the accuracy dropping to 60%.

Both of these recent studies, while demonstrating the promise of deep learning in identifying molecular subtypes of breast cancer, also emphasize the need for further improvement and testing.

In this paper, we study a cutting-edge approach to predict breast cancer molecular subtypes directly from mammogram images. By using a large imaging dataset and advanced AI methodologies, a well-established deep neural network was evaluated. Considering a region of interest containing the tumor, our study includes testing classification strategies (both binary and multi-class), applying different techniques for class balancing and incorporating transfer learning with Resnet-101 to enhance model performance.

## 2. Materials and Methods

### 2.1. Breast Cancer Molecular Subtype Classification

For this study, the information about Ki-67 was not incorporated. Our particular classification on Luminal B1 was: HER2-positive, ER-positive, and PgR negative. In addition, for Luminal B2, only the cases with HER2-positive, ER-positive, and PgR-positive were included.

### 2.2. Database

**Figure 1:**
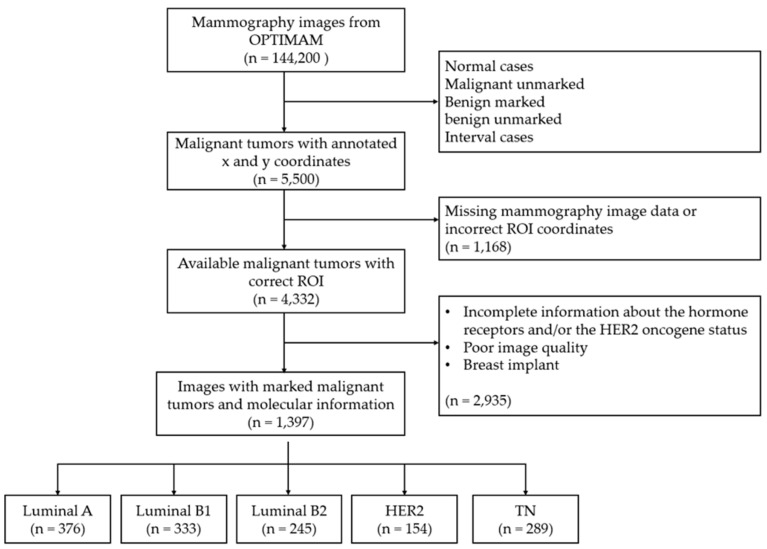
Flowchart of patient inclusion and exclusion criteria in the current study.

This study used mammography images from the OPTIMAM imaging database (OMI-DB) [17,18]. The inclusion criteria required images provided with complete and proven information on the molecular type of breast cancer (based on estrogen and progesterone receptors and the HER2 oncogene), as well as the coordinates of the region of interest (ROI) around the respective tumor. The final dataset of 660 patients (mean age: 67.9 ± 7.7), corresponding to 1397 images of malignant breast tumors of the five pre-defined subtypes: 376 Luminal A, 333 Luminal B, 245 Luminal B2, 154 HER2 and 289 TN, as can be seen in Figure 1.

The dataset used was acquired with equipment from four different manufacturers: 93% from Hologic (Bedford, MA, USA), 3% from Siemens (Siemens AG, Healthcare Sector, Erlangen, Germany), 2% from GE (Madison, WI, USA), and 2% from Philips (Philips Healthcare, Best, The Netherlands).

### 2.3. Convolutional Neural Network (CNN) and Type of Classification

The Resnet-101 deep CNN [19], pre-trained on the ImageNet dataset, which includes approximately 1.2 million images across 1000 classes, was used to evaluate the performance for three types of classification. Binary classifications: where the aim was to distinguish between two classes: (1) each molecular subtype vs. all the others and (2) a direct comparison between two specific molecular subtypes. Multi-class classification: where the possibility of distinguishing all classes of molecular subtype at once is evaluated: Luminal A vs. Luminal B1 vs. Luminal B2 vs. HER2 vs. TN.

### 2.4. Data Processing and Augmentation

**Figure 2:**
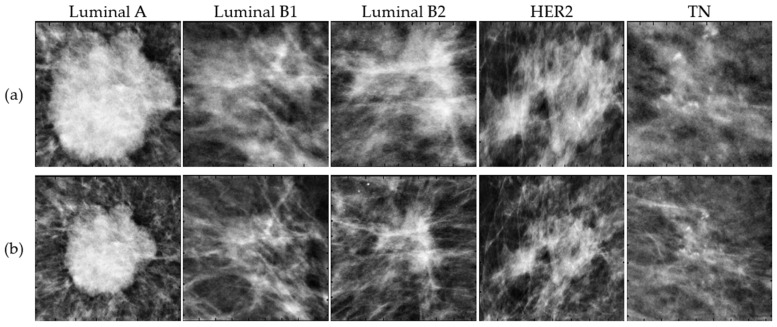
Example of five malignant tumors corresponding to Luminal A, Luminal B1, Luminal B2, HER2 and TN subtypes cropped using the original ROI coordinates (**a**) and larger ROIs (**b**) to contain additional surrounding tissue.

The only processing applied to the mammography images was the cropping of the ROIs based on the coordinates provided. Some ROIs were very close to the tumor and contained little background information about the surrounding breast tissue. In this study, additional information about the tumor’s microenvironment was included since, in recent radiomics studies, this information has proven to be important in the context of tumorigenesis [20,21,22]. Therefore, 100 pixels to the left and right, down and up, were added to each original ROI as shown in Figure 2.

**Figure 3:**
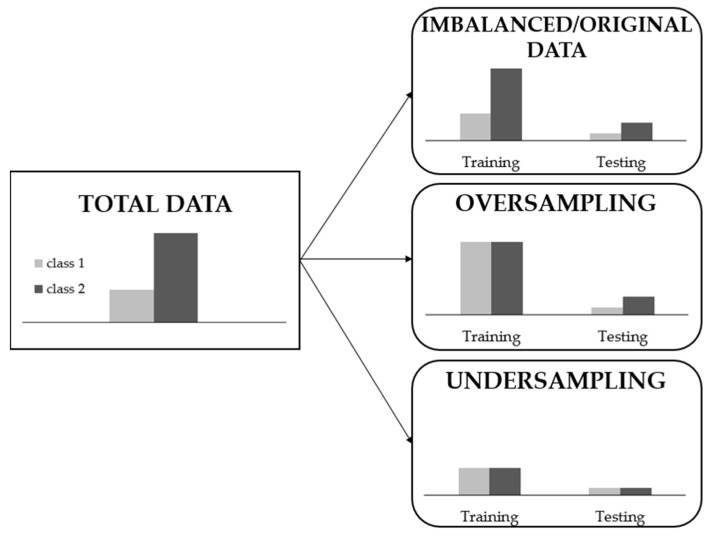
Sampling types for imbalanced data preprocessing considering a binary classification.

Training models with imbalanced data distributions poses significant challenges. However, imbalanced data are common in the majority of real-world scenarios, including our dataset. Solutions for handling imbalanced data can be applied at either the data or algorithmic level. Algorithmic approaches lack generalizability across different datasets as they optimize learning for specific application characteristics [23]. For this reason and considering the imbalance in our classes, the most common data-level solutions were explored as shown in Figure 3:

- *Imbalanced data*: The algorithm was trained using the original dataset distribution for each class, whether binary or multi-class, without any artificial balancing.
- *Oversampling*: During training, we augmented the minority class by artificially increasing its data through random data augmentation, matching the number of samples in the majority class.
- *Undersampling*: We reduced the number of samples in the majority class to match the number of samples in the minority class.

In addition, each of these strategies was evaluated with and without data augmentation applied to all the training data, including random reflections in the left-right and top-bottom directions, rotations between ±20° and horizontal shears between ±10°.

### 2.5. Training Options

The k-fold technique was used as the cross-validation method to estimate the generalization error of the learning process. The dataset was divided into k = 3 subsets, meaning each network was independently trained and tested three times using different subsets of the data. For each fold, 80% of the data was used for training and 20% for testing.

The ResNet-101 was trained using the stochastic gradient descent optimizer with a momentum of 0.9 to minimize the cross-entropy loss for classification. The training was conducted over a maximum of 250 epochs, with a mini-batch size of 64 and a learning rate set to 1 × 10^−4^. To prevent overfitting, an L2 regularization term of 5 × 10^−3^ was incorporated into the loss function.

The artificial intelligence methods under study were implemented using MATLAB R2024a and run on a computer NVIDIA GeForce RTX 4090 GPU (Santa Clara, CA, USA).

### 2.6. Evaluation Metrics

Accuracy and the area under the receiver operating characteristic (ROC) curve (AUC) were the considered metrics to evaluate the Resnet-101 performance. For the binary metric, both metrics were calculated and analyzed, while for the multi-class classification, only the AUC was considered.

In classification tasks, accuracy provides a direct measure of correctly classified samples in relation to the total, but its effectiveness decreases in the presence of unbalanced class distributions, such as this case, providing an incomplete picture of the model’s performance. On the other hand, the AUC assesses the model’s ability to distinguish between positive and negative classes at various threshold values. This evaluation is particularly important in multi-class classifications, where AUC is generally considered the main evaluation metric, offering a comprehensive understanding of the model’s performance across all classes. By considering the full range of classification thresholds, AUC translates the discriminatory power and robustness of the network, ensuring a comprehensive assessment of its performance.

Differences in the performance were tested using a statistical *t*-test. A two-tailed *p*-value < 0.05 was considered to indicate a significant difference.

## 3. Results

### 3.1. Binary Classification: Each Molecular Subtype vs. All the Others

To evaluate the performance of this classification, both accuracy and AUC were calculated. The procedure involved threefold cross-validation, with three different datasets used in each repetition, both for training and testing. Table 2 and Table 3 present the averaged performance and standard deviations for accuracy and AUC, respectively, across the three folds. These results are shown for each classification option, highlighting the different solutions employed to handle imbalanced data.

Table 4 shows the *p*-values calculated to study the measurable statistical differences between the mean accuracies and AUCs found for each classification considering the oversampling with data augmentation solution obtained in Table 2 and Table 3, respectively.

### 3.2. Multi-Class Classification

This classification is similar to the previous one, but it is more complex. Instead of having two classes (subtype vs. non-subtype), we have five distinct classes: Luminal A vs. Luminal B1 vs. Luminal B2 vs. HER2 vs. TN). In other words, the aim here was to identify the exact molecular subtype of breast cancer. Using the same data balancing and data augmentation techniques, the AUC was used to evaluate the Resnet-101 performance (Table 5). As mentioned above, the results are presented considering threefold cross-validation.

The comparison between the AUC values from binary classification (each type versus all others) and those from multi-class classification (distinguishing all types simultaneously) was done based on the statistical analysis presented in Table 6.

**Figure 4:**
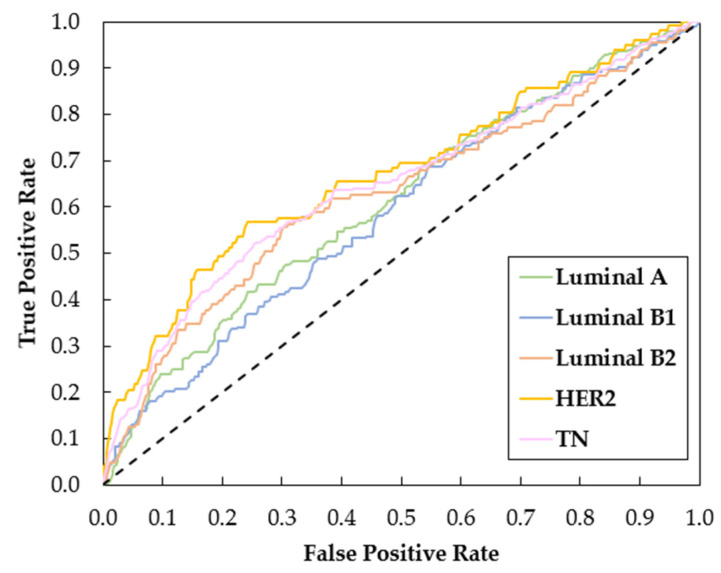
Comparisons of ROC curves in the multi-class classification (Luminal A vs. Luminal B1 vs. Luminal B2 vs. HER2 vs. TN) for the Resnet-101 trained with oversampling and data augmentation.

Considering the results obtained for averaged AUC of oversampling with data augmentation (DA), Figure 4 shows the ROC curves of Resnet-101 trained with the respective data. These curves were obtained by averaging between the ROC curves of each fold.

### 3.3. Binary Classification: Direct Comparison between Two Specific Molecular Subtypes

The aim of this section was to better characterize the intrinsic distinction and relationship between the classification of the different molecular subtypes. To this end, the Resnet-101 was trained for each pair and a direct comparison between two specific molecular subtypes was evaluated. The results are shown in Table 7. For this study, only the oversampling approach with data augmentation was employed, as it consistently demonstrated superior accuracy and AUC compared to previous studies.

**Figure 5:**
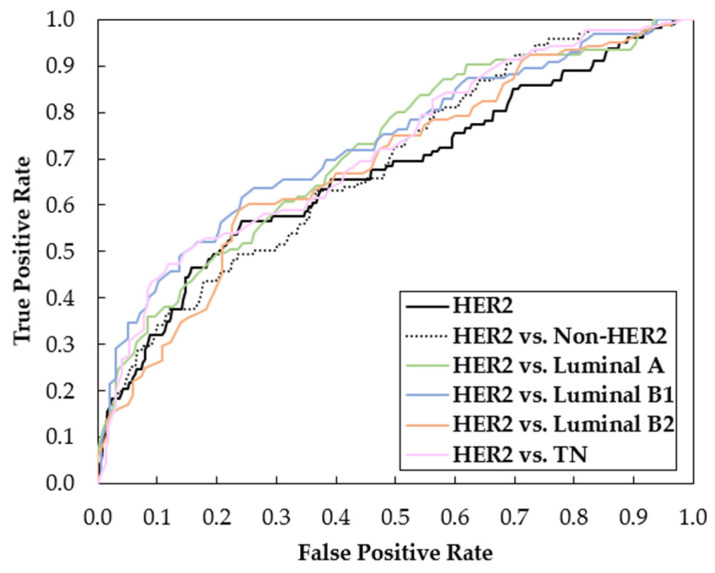
ROC curves obtained using oversampling with data augmentation for classifications involving the HER2 subtype, including HER2 vs. non-HER2 (binary classification), HER2 (multi-class classification) and HER2 vs. each molecular subtype.

Given the promising results for the discrimination of the HER2 subtype, the ROC curves obtained for classifications in which it is involved are represented in Figure 5 (HER2 vs. Non-HER2 from the binary classification of Section 3.1, HER2 from the multi-class classification of Section 3.2 and HER2 vs. each molecular subtype obtained in this section).

## 4. Discussion

In this work, the use of deep learning to predict molecular subtypes of breast cancer from mammogram images using the pretrained Resnet-101 architecture was explored. The approach included binary and multi-class classification scenarios, each addressing various techniques to deal with class imbalance, such as oversampling, undersampling and data augmentation (DA). 

For the binary classification, where one class represented one specific molecular subtype, and the opposite class agglomerated all the others, the results are summarized in Table 2 and Table 3 for the accuracy and AUC, respectively. It is possible to verify that for both the imbalanced and oversampling approaches, all the accuracy values in Table 2 (with and without DA) are above 67%, which indicates a good discriminative performance across all the classes. The undersampling approach, on the other hand, has a relatively poor performance, with results within the range 51.78–62.84%. The AUC results in Table 3, while being relatively lower compared to the accuracy, still represent a fair discriminatory capacity, with some values achieving values higher than 70%. The oversampling approach with DA provided the best performance across subtypes, with an average accuracy of 79.02% (±1.36) and an average AUC of 64.69% (±3.38). This fact demonstrates the importance of balancing techniques in training deep learning models for breast cancer molecular subtype classification.

The classification of HER2 versus non-HER2 achieved the highest average accuracy of 89.79% (±1.27) and AUC at 73.31% (±4.45) across different data balancing strategies, indicating the HER2-positive tumors possess distinct imaging characteristics that the deep learning model can effectively capture. Conversely, the lowest accuracy was noted for Luminal A vs. Non-Luminal A at 51.78% (±3.15), while the lowest AUC was seen in Luminal B1 vs. Non-Luminal B1 classification at 53.11% (±2.14), suggesting that the imaging features of Luminal tumors are less distinct and overlap significantly with other subtypes. 

The statistical analysis (Table 4) revealed significant differences in accuracy between almost all subtypes when using the oversampling with the DA approach. For the AUC values, those differences were not statistically significant.

The multi-class classification aimed to identify the exact molecular subtype among the five categories: Luminal A, Luminal B1, Luminal B2, HER2, and TN. The results (Table 5) showed a more complex scenario compared to the binary classification due to the increased difficulty of the task, as the focus is not only one positive and negative class. For example, for the binary approach, the higher AUC values for Luminal A, Luminal B1, Luminal B2, HER and TN were 65.99, 65.45, 65.30, 73.31 and 64.45, respectively. When doing the multi-class classification, these values drop to 61.01, 59.81, 59.91, 73.14 and 64.27. Those differences were statistically significant in: Luminal A and Luminal B1 with oversampling and DA; Luminal B2 with undersampling and DA; and HER2 using imbalanced data without DA. Only in the latter did multi-classification outperform binary classification. Once again, the HER2 subtype classification highlights from the rest, with its superiority quite visible in the ROC curves in Figure 4.

Regarding these two approaches, it is important to understand how they could impact clinical practice. The binary classification shows better overall results, however, if the case is classified as not being part of the positive class, no more information is obtained regarding the molecular subtype. On the other hand, despite its slightly inferior results, the multi-class classification is promising in terms of identifying the specific molecular subtype, providing more interesting clinical perspectives. 

In order to exploit the benefits of both approaches, Table 7 focuses on the accuracy and AUC results of a binary classification in a one-on-one approach, with all the possible subtypes combined. As it can be seen, the AUC and accuracy values are all above 60%, with the exception of the differentiation between Luminal A and Luminal B1. Once again, corroborating the previously seen results, the classification o HER2 subtype is the one that provides better results with all AUC values near 70%. Taking this into account, Figure 5 shows the ROC curves obtained for the different HER2 subtype classifications using oversampling with DA. In general, the ROC curves obtained with a one-to-one classification showed superior performance, with the HER2 vs. Luminal B1 distinction standing out, as shown by the higher AUC value in Table 7. In addition, it is possible to infer that the multi-class (Figure 5, black solid line) and the binary classification of the HER2 vs. non-HER2 (Figure 5, black dashed line) complement each other. For false positive rates below 0.5, the HER2 classification in the multi-classification approach performs better, while for false positive rates above 0.5, the binary classification stands out.

The ROC curve behavior and the high AUC values obtained for the one-to-one classification approach allow us to reflect on possible methodologies that combine multi-class classification with binary classification. The multi-class approach can be used, for example, to give a broad view regarding the molecular subtype of the analyzed cases. Then, using the classes that achieved higher probabilities, a differential diagnosis can be madeusing the binary classification proposed and depicted in Table 7. An approach like this allows for the benefits of both methodologies to be exploited, obtains information regarding different possible subtypes, then narrows down the possibilities using the top-performing binary classifications.

The use of data balancing techniques, particularly oversampling combined with DA, consistently improved model performance across both binary and multi-class classifications. This approach reduces the impact of class imbalance, a common issue in real-world medical imaging datasets where certain classes are less prevalent. The significant improvement in metrics when applying oversampling with DA suggests that these methods can be used to enhance the model’s ability to generalize and correctly identify minority classes. The poorer undersampling results might be related to the intrinsic characteristics of this method. Since the amount of data is limited by the minority class, removing images from other classes might result in variability loss, impairing the capability of the model to generalize unseen data. 

The ability to accurately classify breast cancer molecular subtypes from mammograms has significant clinical implications. Non-invasive imaging-based classification could complement traditional biopsy methods, providing additional diagnostic information and potentially reducing the need for repeated biopsies. In addition, unlike a biopsy where only a small portion of the tumor is analyzed, imaging gives access to the entire tumor, whether heterogeneous or homogeneous, as well as the surrounding tissue. This approach could streamline the diagnostic process, reduce patient discomfort, and lower healthcare costs associated with invasive procedures.

By utilizing a larger dataset and incorporating deep features through the Resnet-101 architecture, we demonstrate the feasibility of achieving higher accuracy and AUC values in the prediction of molecular breast cancer subtypes directly from mammography images. Despite the promising results, our study has limitations. The exclusion of Ki-67 information, which is an important marker for breast cancer classification, may have impacted the model’s ability to fully distinguish between subtypes. Furthermore, the class imbalance, while addressed through the mentioned techniques, still requires continuous refinement. Subtypes such as TN and HER2 were underrepresented compared to Luminal A, affecting the model’s performance and generalizability. Directly related to this limitation, is the DA techniques used. DA represents a valuable resource in a context of data sparsity and can be of extreme importance to increase model’s ability to generalize to unseen data. However, the techniques used were based on geometric transformations, which may limit the variability of the data used for training. Given that, future work should focus on more sophisticated balancing techniques, such as synthetic minority over-sampling (SMOTE) or generative adversarial networks (GANs), that are capable of creating new images with relatively low input data. On the other hand, while deep learning models like Resnet-101 can capture complex features, the representation of subtle differences between molecular subtypes remains challenging. The integration of more advanced feature extraction techniques and the incorporation of additional clinical and genetic data could enhance model performance. Transfer learning with more diverse pre-trained models should also be explored.

The average age of the women included in our study was 67.9 (±7.7), with a minimum of 50 and a maximum of 90 years old. Age is an important factor in the detection of breast cancer in general and in predicting the molecular subtype in particular, essentially because of two factors: breast density and hormone levels. In both cases, there are changes depending on age. For younger women, we are generally dealing with higher breast densities, which can make it more challenging to predict the subtype using mammography images (which very often result in the superposition of the tissues). On the other hand, younger women are more likely to develop aggressive types like TN, while older women are more likely to develop ER+ and PR+ cancers. With regard to density, we had no information about the participants’ breast density at the time this study was carried out. As for the hormonal levels, despite the imbalance classes and the average age of 67.7, the dataset corresponds to a very heterogeneous group and all the significant types have been included. Regardless, in order to ensure better generalizability, it is our aim in the future to introduce data from other databases (and even other modalities, such as breast tomosynthesis) with different age ranges and known density information.

## 5. Conclusions

Our study demonstrates the potential of deep learning models, specifically the Resnet-101 architecture, to predict breast cancer molecular subtypes from mammogram images, highlighting the importance of data balancing and augmentation techniques. Despite the challenges, the promising results pave the way for further research and development in this field, with the aim of improving personalized treatment strategies and non-invasive diagnostic approaches for breast cancer patients.

## Figures and Tables

**Table 1:** Definitions of intrinsic subtypes of breast cancer classification from the St. Gallen Consensus 2013 [4]. HER2, human epidermal growth factor receptor 2; ER, estrogen receptors; PgR, progesterone receptors.

Intrinsic Subtype	HER2	ER	PgR	Ki-67
Luminal A	Negative	Positive	Positive	Low
Luminal B1	Negative	Positive	Negative or Ki-67 high	
Luminal B2	Positive	Positive	Any	Any
HER2 enriched (“HER2”)	Positive	Negative	Negative	Any
Triple Negative (“TN”)	Negative	Negative	Negative	any

**Table 2:** Performance of Resnet-101 trained with imbalanced data, oversampling, and undersampling (without and with Data Augmentation (DA)) in classifying Luminal A, Luminal B1, Luminal B2, HER2, and TN versus non-respective classes, in terms of accuracy. * *p* < 0.05: There is a significant difference between groups. In the line of average values, the best value obtained is highlighted in bold.

	Accuracy (%): Mean (stdDev)																							
	Imbalanced Data								Oversampling								Undersampling							
	without DA				with DA				without DA				with DA				without DA				with DA			
**Luminal A vs. Non-Luminal A**	72.28 (1.80)				67.14 (2.04)				70.73 (2.72)				73.72 (0.74)				51.78 (3.15)				60.00 (5.93)			
**Luminal B1 vs. Non-Luminal B1**	74.08 (1.36)				71.21 (1.81)				73.95 (0.41)				74.91 (1.99)				55.14 (4.89)				55.14 (7.22)			
**Luminal B2 vs. Non-Luminal B2**	79.33 (2.71)				78.14 (0.95)				79.69 (2.27)				80.76 (1.66)				58.07 (2.59)				61.17 (2.38)			
**HER2 vs. Non-HER2**	88.29 (1.66)				89.79 (1.27)				88.77 (1.36)				88.65 (1.80)				59.02 (5.91)				62.84 (8.25)			
**TN vs. Non-TN**	77.42 (2.18)				73.96 (2.90)				76.70 (2.24)				77.06 (0.62)				59.13 (5.29)				55.65 (4.52)			
**Average**	78.28 (1.94)				76.05 (1.79)				77.97 (1.80)				**79.02 (1.36)**				56.63 (4.37)				58.96 (5.66)			
																			*					
																	*							
															*									
			*																					

**Table 3:** Performance of Resnet-101 trained with imbalanced data, oversampling, and undersampling (without and with Data Augmentation (DA)) in classifying Luminal A, Luminal B1, Luminal B2, HER2, and TN versus non-respective classes, in terms of AUC. * *p* < 0.05: There is a significant difference between groups. In the line of average values, the best value obtained is highlighted in bold.

	AUC (%): Mean (stdDev)																							
	Imbalanced Data								Oversampling								Undersampling							
	without DA				with DA				without DA				with DA				without DA				with DA			
**Luminal A vs. Non-Luminal A**	62.04 (6.91)				61.48 (1.59)				59.42 (1.46)				65.99 (1.30)				53.29 (4.94)				65.70 (5.15)			
**Luminal B1 vs. Non-Luminal B1**	57.10 (1.26)				62.82 (1.11)				53.11 (2.14)				65.45 (2.50)				57.81 (4.30)				56.74 (6.88)			
**Luminal B2 vs. Non-Luminal B2**	57.65 (2.33)				59.07 (4.23)				56.45 (3.07)				58.85 (3.98)				64.48 (4.20)				65.30 (3.00)			
**HER2 vs. Non-HER2**	61.33 (1.51)				71.35 (4.80)				65.20 (4.04)				68.71 (6.89)				63.38 (8.74)				73.31 (4.45)			
**TN vs. Non-TN**	56.43 (4.36)				59.96 (0.59)				56.16 (5.62)				64.45 (2.22)				60.82 (6.16)				57.66 (4.20)			
**Average**	58.91 (3.27)				62.94 (2.46)				58.07 (3.27)				**64.69 (3.38)**				59.96 (5.67)				63.74 (4.74)			
													*											
									*															

**Table 4:** Levels of significance (*p*-values) obtained from the statistical analysis of the difference between the mean accuracies and AUCs found for each classification considering the oversampling with data augmentation solution. The results are expressed in terms of accuracy/AUC. *p* < 0.05 is represented in bold: There is a significant difference between groups.

	Luminal B1 vs. Non-Luminal B1	Luminal B2 vs. Non-Luminal B2	HER2 vs. Non-HER2	TN vs. Non-TN
**Luminal A vs. Non-Luminal A**	0.415/0.761	**0.009**/0.078	**0.002**/0.567	**0.004**/0.370
**Luminal B1 vs. Non-Luminal B1**	-	**0.019**/0.084	**0.001**/0.507	0.196/0.632
**Luminal B2 vs. Luminal B2**	-	-	**0.005**/0.115	**0.047**/0.119
**HER2 vs. Non-HER2**	-	-	-	**0.004**/0.399

**Table 5:** Performance of Resnet-101 trained with imbalanced data, oversampling, and undersampling (without and with Data Augmentation (DA)) in the multi-class classification of Luminal A vs. Luminal B1 vs. Luminal B2 vs. HER2 vs. TN, in terms of AUC. For each molecular subtype, the best AUC value obtained is shown in bold.

	AUC (%): Mean (stdDev)					
	Imbalanced Data		Oversampling		Undersampling	
	without DA	with DA	without DA	with DA	without DA	with DA
**Luminal A**	**61.01 (4.53)**	59.92 (2.65)	57.53 (4.66)	54.09 (1.80)	53.11 (3.96)	58.05 (6.23)
**Luminal B1**	55.52 (3.55)	**59.81 (2.09)**	51.00 (9.50)	53.89 (3.14)	58.16 (3.91)	56.13 (2.87)
**Luminal B2**	56.43 (0.86)	59.20 (4.22)	52.09 (7.08)	**59.91 (0.28)**	57.65 (5.88)	53.36 (2.57)
**HER2**	66.27 (2.40)	64.40 (2.40)	**73.14 (4.38)**	70.96 (4.00)	70.01 (4.34)	66.78 (4.19)
**TN**	58.06 (1.98)	60.89 (5.51)	53.39 (1.72)	**64.27 (2.26)**	57.70 (9.44)	61.06 (2.60)
**Average**	59.46 (2.66)	**60.84 (3.37)**	57.43 (5.47)	**60.62 (2.30)**	59.33(5.51)	59.08 (3.69)

**Table 6:** Levels of significance (*p*-values) obtained from the statistical analysis of the difference between the mean AUCs found for each type of classification (binary and multi-classification). *p* < 0.05 is represented in bold: there is a significant difference between classifications.

	Imbalanced Data		Oversampling		Undersampling	
	without DA	with DA	without DA	with DA	without DA	with DA
Luminal A vs. Non-Luminal A	0.843	0.442	0.562	**0.001**	0.963	0.225
Luminal A (multi-classification)						
Luminal B1 vs. Non-Luminal B1	0.532	0.113	0.741	**0.009**	0.923	0.899
Luminal B1 (multi-classification)						
Luminal B2 vs. Non-Luminal B2	0.470	0.971	0.407	0.691	0.184	**0.007**
Luminal B2 (multi-classification)						
HER2 vs. Non-HER2	**0.049**	0.112	0.082	0.655	0.326	0.138
HER2 (multi-classification)						
TN vs. Non-TN	0.600	0.798	0.488	0.929	0.661	0.311
TN (multi-classification)						

**Table 7:** Averaged performance of Resnet-101 for each paired classification in terms of accuracy and AUC, trained with oversampling and data augmentation (mean (standard deviation) considering the threefold cross-validation). The maximum AUC values obtained are shown in bold.

	Accuracy (%)	AUC (%)
**HER2 vs. Luminal A**	**76.42 (1.89)**	71.54 (2.80)
**HER2 vs. Luminal B1**	74.91 (4.17)	**73.04 (1.68)**
**HER2 vs. Luminal B2**	64.56 (1.27)	68.62 (3.58)
**HER2 vs. TN**	71.97 (5.12)	71.66 (3.25)
**Luminal A vs. Luminal B1**	56.97 (2.86)	59.35 (3.94)
**Luminal A vs. Luminal B2**	62.10 (3.70)	65.81 (7.20)
**Luminal A vs. TN**	61.41 (6.82)	63.90 (5.99)
**Luminal B1 vs. Luminal B2**	62.90 (1.33)	65.88 (3.39)
**Luminal B1 vs. TN**	61.29 (2.91)	64.59 (2.69)
**Luminal B2 vs. TN**	63.21 (1.89)	68.22 (2.42)

## Data Availability

The dataset presented in this article is not readily available. Requests to access the datasets should be directed to the OPTIMAM providers.
